# Terrestrial *Trentepohlia* sp. (Ulvophyceae) from alpine and coastal collection sites show strong desiccation tolerance and broad light and temperature adaptation

**DOI:** 10.1007/s00709-023-01866-2

**Published:** 2023-06-08

**Authors:** Andreas Holzinger, Niklas Plag, Ulf Karsten, Karin Glaser

**Affiliations:** 1 Department of Botany, University of Innsbruck, Sternwartestrasse 15, 6020 Innsbruck, Austria; 2 Applied Phycology and Ecology, University of Rostock, Albert Einstein Strasse 3, 18059 Rostock, Germany

**Keywords:** *Trentepohlia* sp., Desiccation tolerance, Light and temperature adaptation, Aerophytic biofilm, Cell wall staining, Compatible solutes

## Abstract

For the present study, we collected the Ulvophyceae species *Trentepohlia aurea* from limestone rock near Berchtesgaden, Germany, and the closely related taxa *T. umbrina* from *Tilia cordata* tree bark and *T. jolithus* from concrete wall both in Rostock, Germany. Freshly sampled material stained with Auramine O, DIOC_6_, and FM 1–43 showed an intact physiological status. Cell walls were depicted with calcofluor white and Carbotrace. When subjected to three repeated and controlled cycles of desiccation over silica gel (~ 10% relative humidity) followed by rehydration, *T. aurea* recovered about 50% of the initial photosynthetic yield of photosystem II (YII). In contrast, *T. umbrina* and *T. jolithus* recovered to 100% of the initial YII. HPLC and GC analysis of compatible solutes found highest proportions of erythritol in *T. umbrina* and mannitol/arabitol in *T. jolithus*. The lowest total compatible solute concentrations were detected in *T. aurea*, while the C/N ratio was highest in this species, indicative of nitrogen limitation. The prominent orange to red coloration of all *Trentepohlia* was due to extremely high carotenoid to Chl *a* ratio (15.9 in *T. jolithus*, 7.8 in *T. aurea*, and 6.6. in *T. umbrina*). Photosynthetic oxygen production was positive up to ~ 1500 µmol photons m^−2^ s^−1^ with the highest P_max_ and alpha values in *T. aurea*. All strains showed a broad temperature tolerance with optima for gross photosynthesis between 20 and 35 °C. The presented data suggest that all investigated *Trentepohlia* species are well adapted to their terrestrial lifestyle on exposed to sunlight on a vertical substrate with little water holding capacity. Nevertheless, the three *Trentepohlia* species differed concerning their desiccation tolerance and compatible solute concentrations. The lower compatible solute contents in *T. aurea* explain the incomplete recovery of YII after rehydration.

## Introduction

The typical orange to red macroscopic appearance of several *Trentepohlia* sp., members of the green algal class Ulvophyceae, is a well-known phenomenon, as this genus occurs on natural habitats like bark or rocks (e.g., Liu et al. 2012, as well as on man-made surfaces like concrete walls (e.g., Rindi and Guiry 2002, Rindi et al. 2009). The terrestrial lifestyle can be considered as quite rare amongst Ulvophyceae. Most Trentepohliales are free-living, yet lichen forming members are described (Hametner et al. 2014).

While several studies have dealt with molecular-taxonomic aspects of the genus *Trentepohlia* (Rindi and López-Bautista 2007, López-Bautista et al. 2006; Rindi et al. 2005; Bartoli et al. 2019; Klimesová et al. 2019), comparably little is known on their biochemistry, ecophysiology, and molecular biology (e.g., Ong et al. 1992; Tan et al. 1993; Li et al. 2014; Zhang et al. 2016, 2020; Garcia-Florentino et al. 2018; Ritchie and Heemboo 2021) or subcellular organization (Chapman et al. 2001; López-Bautista et al. 2003). Astonishingly, it has been shown that the cytokinesis of *Trentepohlia* is phragmoplast-mediated, a type of cytokinesis that is usually known from land plants and some streptophytic algae (Buschmann and Zachgo 2016). Even indications for the occurrence of phragmoplastin, a dynamin-like protein that has been associated with cell plate formation has been detected by PCR amplification in *Trentepohlia odorata* and *Cephaleuros parasiticus*, another subaerial green alga within the Trentepohliales (López-Bautista et al. 2003). These findings are very exceptional, as Trentepholiales are members of the class Ulvophyceae within the Chlorophyta where in most cases phycoplasts occur.

The orange-red pigments of this green alga were first researched in the late nineteenth century, when carotenoids were found, which were comparable with those of carrot (Zopf 1892). Later, α- and β-carotene from *Trentepohlia* sp. were isolated and identified as zeaxanthin and lutein as well as an additional carotenoid detected, which turned blue when hydrochloric acid was added (Tischer 1936; Kjosen et al. 1972; Nybraate and Liaaen-Jensen 1974). The carotenoids of *Trentepohlia* have been further characterized by Kharkongor and Ramanujam (2015) and a comparative study on carotenoid accumulation in *T. arborum* as a consequence of different culture condition has been performed by Chen et al. (2016). Mukherjee et al. (2010) reported on the seasonal variations in carotenoid content of *T. aurea* and *T. cucullata*. Thus, several studies have addressed the most prominent visible feature of *Trentepohlia*, the high carotenoid content (Tan et al. 1993; Chen et al. 2016; Kharkongor and Ramanujam 2015). A study on the chemical characterization of unusual carbohydrate patterns of different *Trentepohlia* species was performed by Feige and Kremer (1980), who detected a variety of polyols that have a putative function as compatible solutes in *Trentepohlia*.

As *Trentepohlia* sp. are exposed in their natural habitats to varying water conditions, they show an extreme form of desiccation tolerance, and the phenomenon is macroscopically visible when they grow on bare rock or concrete walls (e.g., Rindi and Guiry 2002). Yet, to our knowledge, only few studies have addressed desiccation tolerance in *Trentepohlia* (Ong et al. 1992; Zhang et al. 2016, 2020) and one study exists on the effect of osmotic stress in *Trentepohlia* (Ritchie and Heemboo 2021). Zhang et al. (2016) demonstrated in *T. jolithus* under controlled desiccation that the efficiency of PSII light absorption and activities of PSII reaction centers were reversibly down-regulated, which was interpreted as a special adaptive mechanism for survival under terrestrial conditions. In a later study, Zhang et al. (2020) provided more mechanistic understanding on the desiccation tolerance in *T. jolithus* by demonstrating that the non-photochemical quenching (NPQ) gradually decreased during desiccation, while the photochemical efficiency of photosystem II (ΦPSII) remained unchanged. These data indicate that the photoprotective mechanism NPQ was more sensitive to desiccation than photosynthetic linear electron flow. However, in strongly desiccated cells of *T. jolithus* some key enzymes of the Calvin cycle were activated and thus promoting the rapid recovery of linear electron flow after rehydration (Zhang et al. 2020). Consequently, the authors argued that a combination of mechanisms explain the high desiccation tolerance in *T. jolithus*, i.e., the reversible down-regulation of reaction centers and the maintenance of weakened, but still linear electron flow during desiccation, and the rapid recovery of the original linear electron flow of desiccated algal cells.

Ritchie and Heemboo (2021) exposed *Trentepohlia* cells to 1.5 Osmol kg^−1^ mannitol and found that photosynthesis was highly resistant to this treatment and cells could recover quickly from the osmotic stress. It has to be stated that mannitol might not have been the best selection as osmotically active substance, because this polyol naturally occurs in *Trentepohlia* (Feige and Kremer 1980) and thus might be metabolized during the experiments. In the study by Ong et al. (1992), *T. odorata* has been exposed to 100%, 75%, and 43% relative humidity (RH) for up to 7 days, leading to a decrease in carotenoid to chlorophyll content and changes in photosynthetic parameters. In general, desiccation tolerance, i.e., the capacity to survive drying below 65% relative humidity (RH), corresponding to an absolute water content of 0.1 g H_2_O g^–1^ dry mass and or a water potential of <  − 100 MPa (Oliver et al. 2020) can be examined by actually exposing cells to air of a defined relative humidity. Desiccation tolerance has recently been investigated in various green algae which showed different recovery rates depending on the RHs they have been exposed to (e.g., Aigner et al. 2020; Karsten et al. 2014; Roach et al. 2022; Terlova et al. 2021). Yet, for *Trentepohlia* sp. such experiments are still rare (Zhang et al. 2016, 2020). Also, a comprehensive ecophysiological characterization concerning light- and temperature demands for photosynthesis has not been performed yet. In addition, some terrestrial algae form resting cells or cysts as an adative trait which guarantees survival under long term unfavourable environmental conditions (Ettl and Gärtner 2014).

The aim of the present study was to characterize field collected *Trentepohlia* sp. samples from different origins by vital and cell wall straining, as well as to analyze their desiccation tolerance and physiological requirements concerning light and temperature optima. We hypothesized that different species, known to have particular habitat preferences show differences in desiccation tolerance and/or key polyols involved in desiccation tolerance. Our second hypothesis was that species from alpine and coastal origin respond differently concerning their light- and temperature demands for photosynthesis. In order to test these hypotheses, we collected three different *Trentepohlia* species namely, *T. aurea* from a natural habitat at limestone in an alpine region and *T. umbrina* from tree bark and *T. jolithus* from a concrete wall in a coastal region, and performed a standardized set of physiological experiments combined with a microscopic examination of the field collected samples and chemical analysis of their C/N content and compatible solutes.

## Material and methods

### Algal material

Three different *Trentepohlia* sp. were collected in early summer 2022: (1) *Trentepohlia aurea* (L.) C. Martius on limestone rock near Bad Reichenhall, Germany (N47°70.043 E12°85.187) two specimens were collected in Rostock: (2) *Trentepohlia umbrina* (Kützing) Bornet on bark of a *Tilia cordata* tree in Rostock, Kröpeliner Tor Vorstadt (N54°05.161 E12°06.652), and (3) *Trentepohlia jolithus* (L.) Wallr. on a concrete wall in the Rostock Harbor (N54°05.598 E12°08.696). Bad Reichenhall has a cold-temperate climate and is characterized as (Dfb: winter-wet-cold with warm summer) according the Köppen and Geiger (1930-1939) classification. The average annual temperature is 6.7 °C with an annual precipitation of 1870 mm (www.climate-data.org). In contrast Rostock has a temperate climate and is characterized as (Cfb: humid-temperate with warm summer) according the Köppen and Geiger (1930-1939) classification. The average annual temperature is 9.6 °C with an annual precipitation of only 730 mm (www.climate-data.org).

All physiological experiments were performed with freshly collected field material and additionally, field material was dried at 40 °C for extended storage.

### Light and fluorescence microscopy

*Trentepohlia aurea*, *T. umbrina*, and *T. jolithus* cells were stained with 0.1% Auramine O (Sigma-Aldrich, Steinheim, Germany), 1% Calcofluor white (Sigma-Aldrich, Steinheim, Germany; catalog number: 18909) or Carbotrace 480 (blue), Carbotrace 520 (green) and Carbotrace 540 (yellow) (Ebba Biotech AB, Solna, Sweden) for cell wall staining. Images were taken on an Olympus BX-51 microscope (Olympus, Tokyo, Japan) equipped with an Olympus UC30 digital camera (Olympus, Tokyo, Japan). The microscope is equipped with different filter sets for fluorescence microscopy (WB, WG, WU, and WIB).

### Confocal laser scanning microscopy

*Trentepohlia aurea*, *T. umbrina*, and *T. jolithus* cells stained with ~ 1 mg ml^−1^ DIOC_6_ or 20 µM FM 1–43 dye (prepared from a 20 mM stock in dimethylsulfoxide (DMSO) according to Holzinger et al. (2011) or 1 µM Nile red (from 1 mM stock in DMSO) were investigated by confocal laser scanning microscopy (CLSM, Leica SP-1, Leica Microsystems, Mannheim, Germany). Excitation was generated with an argon laser at 488 nm, emission was collected in the green rage from 500 to ~ 560 nm, and in the red range with a longpass of 580 nm. Images were false colored green and red, further processed with ImageJ (Fiji).

### Photosynthetic oxygen production in dependence of light and temperature

Photosynthetic oxygen evolution rates of *T. aurea, T. umbrina*, *and T. jolithus* were measured with a Presens Fibox3 oxygen optode (Presens, Germany) either under (1) increasing photon flux densities from 0 to 1580 μmol photons m^−2^ s^−1^ at 20 °C or under (2) increasing temperatures from 5 to 45 °C according to Karsten et al. (2010) and Prelle et al. (2019). For the photosynthesis-irradiance curves measurements started with a respiration phase of 30 min in the dark followed by a 10–15-min photosynthesis phase for each of the continuously rising light levels. For temperature curves, cell suspensions were incubated for 10 min under darkness followed by 30-min incubation at ~ 300 µmol photons m^−2^ s^−1^ in a series of 4 × 3 mL custom built thermostatic acrylic chambers combined with magnetic stirrers (Hansatech Instruments, UK). The O_2_ production was normalized to the amount of total chlorophyll *a* (Chl *a*) per sample. After photosynthesis-irradiance (P-I) curve or temperature curve measurements, the cell suspension was filtered onto Whatman GF 6 glass fiber filters (Whatman, Dassel, Germany). Chl *a* was extracted with 100% ethanol (v/v) for 30 min at 78 °C, followed by 10 min over ice, samples were then centrifuged and quantified according to Ritchie (2006). P-I curves were calculated and fitted by the mathematical photosynthesis model of Walsby (1997) with the SOLVER-function from Excel (Microsoft Office 365) which allowed the calculation of the three parameters: α (positive slope at limiting photon flux densities), I_C_ (light compensation point), and I_K_ (initial value of light-saturated photosynthesis). Temperature curve data were fitted with and further processed by the model of Yan and Hunt (1999) implemented in R.

### Desiccation experiment

*Trentepohlia aurea*, *T. umbrina*, and *T. jolithus* were subjected to repeated desiccation and rehydration cycles in a previously described desiccation chamber (Karsten et al. 2014). In one of these chambers three GF filters, with four specimens of biomass per filter were placed, giving a total of 12 specimens (*n* = 12). The filters were kept under continuous low-light conditions (40–50 μmol photons m^−2^ s^−1^) conditions. Freshly dried silica gel was used as desiccant, creating a relative humidity of ~ 10% in the chambers and the effective quantum yield of photosystem II (YII) was monitored in a defined distance (1 cm above the specimen) from the outside of the chambers with a PAM 2500 (Walz, Effeltrich, Germany) applying actinic light pulses of 1200–1500 μmol photons m^−2^ s^−1^. Specimens were desiccated until all four samples reached YII of zero. For visualization of the desiccation effect, small portions of desiccated samples dried over silica gel at ~ 10% RH were prepared in immersion oil for microscopic observations in an Olympus BX-51 microscope (Olympus, Tokyo, Japan). For rehydration, the filters were placed in a chamber filled with distilled water (producing a RH of about ~ 95%) instead of desiccant and the filters were soaked with 400 µL BBM, but it was taken care that the sample was not directly wetted.

### Spectrophotometric pigment determination

To measure pigments in *Trentepohlia* 10 mg dry biomass was incubated with 3 mL 100% dimethylformamid at 5 °C overnight. Afterwards, the samples were centrifuged at 3000 × g for 1 min. The solution was of visually red color and had to be diluted. Measurement was conducted using a UV/Vis spectrophotometer (UV/VIS spectrometer Shimadzu UV-2401 PC, Kyoto, Japan). The amount of carotenoid and chlorophyll was calculated after Wellburn (1994) using wavelengths at 480 nm, 647 nm, 663 nm, and 750 nm.

#### Determination of C/N content

To estimate the C and N content of *Trentepohlia* biofilms, all samples were dried at 40 °C overnight. Afterwards, three replicates of 300 mg ground biomass per sample was analyzed with an Elementar Unicube (Elementar, Langensbold, Germany). Whereas *T. aurea* and *T. jolithus* contained mostly pure biomass, due to the inorganic substrata they were growing on, in the case of *T. umbrina* also carbon intake from the bark of the *Tilia cordata* tree cannot be excluded.

### Compatible solute detection by HPLC and GC analysis

For polyol extraction 10 mg of dry material of *T. aurea*, *T. umbrina*, and *T. jolithus* was extracted in 70% ethanol at 70 °C for 4 h. After centrifugation at 8450 × g for 5 min, 800 µL supernatant was evaporated and dissolved in the same volume of autoclaved ultrapure water (55 µS cm^−1^). After further centrifugation steps, the supernatant was stored in a 2-mL screw cap glass vial with silicone/PTFE septum (Wicom, Heppenheim, Germany) and frozen at − 18 °C until final analysis.

High performance liquid chromatography (HPLC) was performed on an Agilent 1260 Infinity HPLC system with RI-detector (Agilent Technologies, Santa Clara, CA, USA). Fast Carbohydrate Column (Bio-Rad, Feldkirchen, Germany) with a Carbo-Pb^2+^ guard column (Phenomenex, Aschaffenburg, Germany) were used for separation at 70 °C and a flow rate of 1.0 mL min^−1^ at a pressure of 45 bar. As calibration substances Adonitol, D(-)-Mannitol, D( +)-Arabitol, meso-Eryhtritol, Glycerol (all Carl Roth, Karlsruhe, Germany) and for volemitol an extract of *Pelvetia canaliculata* were used. Due to the same retention time of arabitol and mannitol in the HPLC analysis, it was not possible to distinguish between these two polyols in the samples.

Gas chromatography (GC) was used to re-examine the results from HPLC. For preparation, polyol extracts and standards were derivatized with 300 µl Pyridine (Merck, Darmstadt, Germany) and 300 µL N,O-Bis(trimethylsilyl)trifluoroacetamide (Merck, Darmstadt, Germany) while shaking for 1 h at 60 °C. The analysis was executed on an Agilent Technologies G1530N Network GC System with a G2614A Autosampler and G2613A Injector (Agilent Technologies, CA, USA). Separation of the polyols was performed with a TG-5MS column (Thermo Fisher Scientific, Dreieich, Germany), at an inlet temperature of 280 °C, split flow was at 11 mL min^−1^, column flow was set to 1.8–1.9 mL min^−1^, resulting in a velocity of 35–40 cm s^−1^. At a speed of 25 mL min^−1^, nitrogen-gas was used as makeup gas and 40 mL min^−1^ hydrogen gas mixed with 400 mL min^−1^ compressed air was used as burning fuel. Polyol proportions given by GC analysis were applied on the data of HPLC analysis to calculate the concentration of arabitol and mannitol.

### Statistical analysis

All statistics and analyses were done in R software (version 4.2.1; R Core Team (2019)) or using Excel solver function. Fitting of PI curve according the Walsby Model was done using the solver function in Excel; fitting of temperature curve according to Yan and Hunt model (Yan and Hunt 1999) was done in R. Significant differences between points were calculated using Anova followed by Tukey *post hoc* analysis implemented in R.

## Results

### Macroscopic and microscopic appearance of the investigated strains

**Fig. 1:**
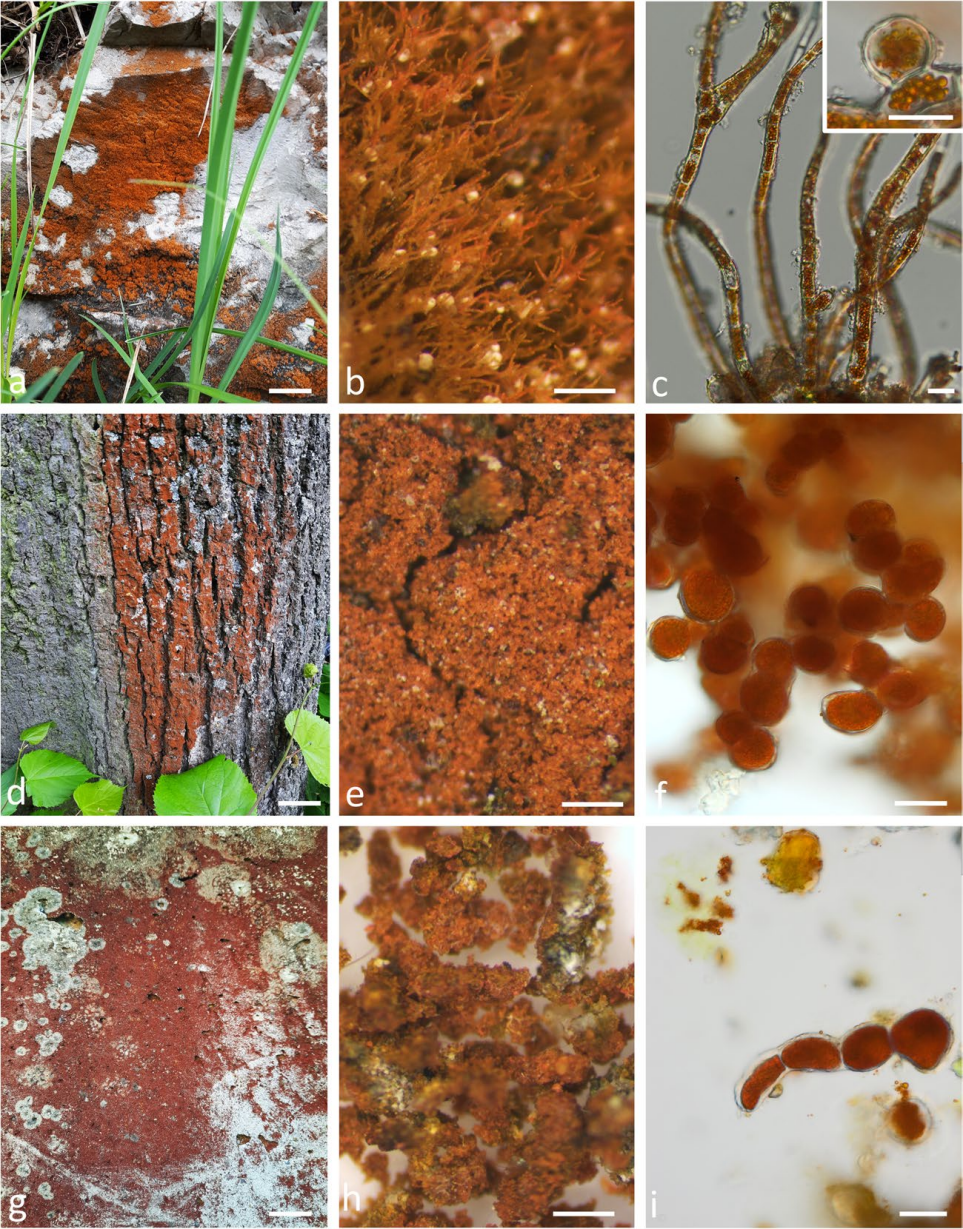
Characterization of habitat, macroscopic view by stereo microscope and light microscopic appearance of *Trentepohlia aurea* collected from limestone (**a**–**c**), insert in **c** showing a sporangium, *Trentepohlia umbrina* collected from *Tilia cordata* bark (**d**–**f**) and *Trentepohlia jolithus* collected from concrete wall (**g**–**i**). Scale bars** a**, **d**, **g**: 2 cm; **b**, **e**, **h**: 400 µm; **c**, **f**, **g**: 20 µm

*Trentepohlia aurea* was collected from limestone and had an orange coloration (Fig. 1a) exhibiting under the stereo microscope a trichome-like appearance (Fig. 1b), with branched filaments (Fig. 1c) occasionally showing sporangia (Fig. 1c, inset). The cells of *T. aurea* had an average diameter of 13.6 ± 2.6 µm and an average length of 29.7 ± 5.7 µm (*n* = 14). *Trentepohlia umbrina* was collected from *Tilia cordata* bark in areas where it was abundant (Fig. 1d) and showed granular appearance in the stereo microscope (Fig. 1e), and were composed of packages of several globose cells (Fig. 1f). The cells exhibited an average diameter of 16.1 ± 3.1 µm (*n* = 14). *Trentepholia jolithus* cells were collected from a concrete wall (Fig. 1g), and the cells attached to wall debris had a granular appearance (Fig. 1h). Cell aggregates contained several filamentous assemblies of 4–5 cells, but the filament length was likely reduced by harvesting with a knife. The basal cells were usually thinner in diameter (Fig. 1i) and cells showed an average diameter of 19.4 ± 1.8 µm (*n* = 14).

### Viability tests, lipid, and cell wall staining

**Fig. 2:**
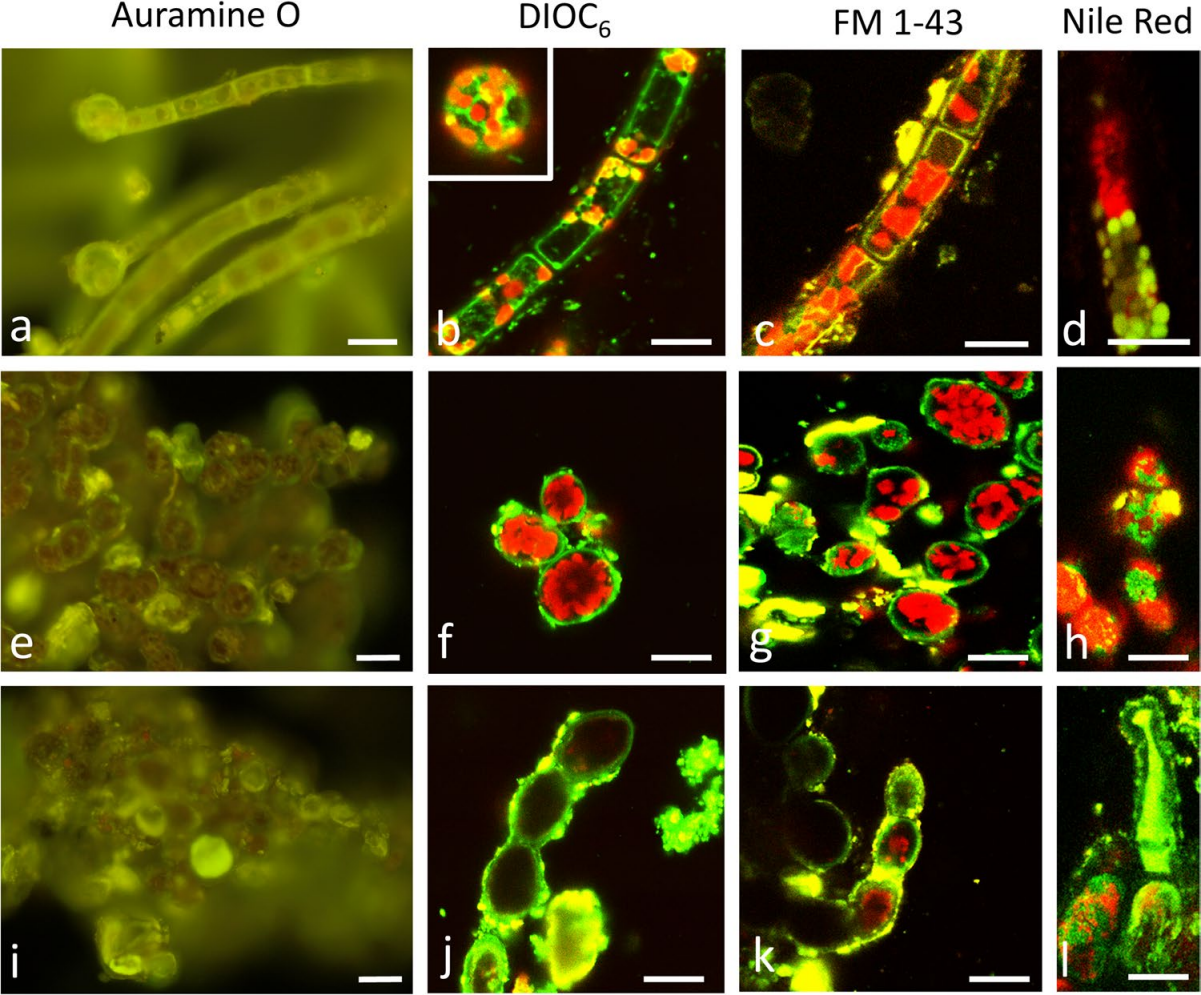
*Trentepohlia aurea* (**a**–**d**), *Trentepohlia umbrina* (**e**–**h**), *Trentepohlia jolithus* (**i**–**l**) stained with Auramine O (**a**, **e**, **i**) observed by fluorescence microscopy (filter WIB), DIOC_6_ (**b**, **f**, **j**), FM 1–43 (**c**, **g**, **k**) or Nile red (**d**, **h**, **l**) observed by confocal laser scanning microscopy. Scale bars 20 µm

**Fig. 3:**
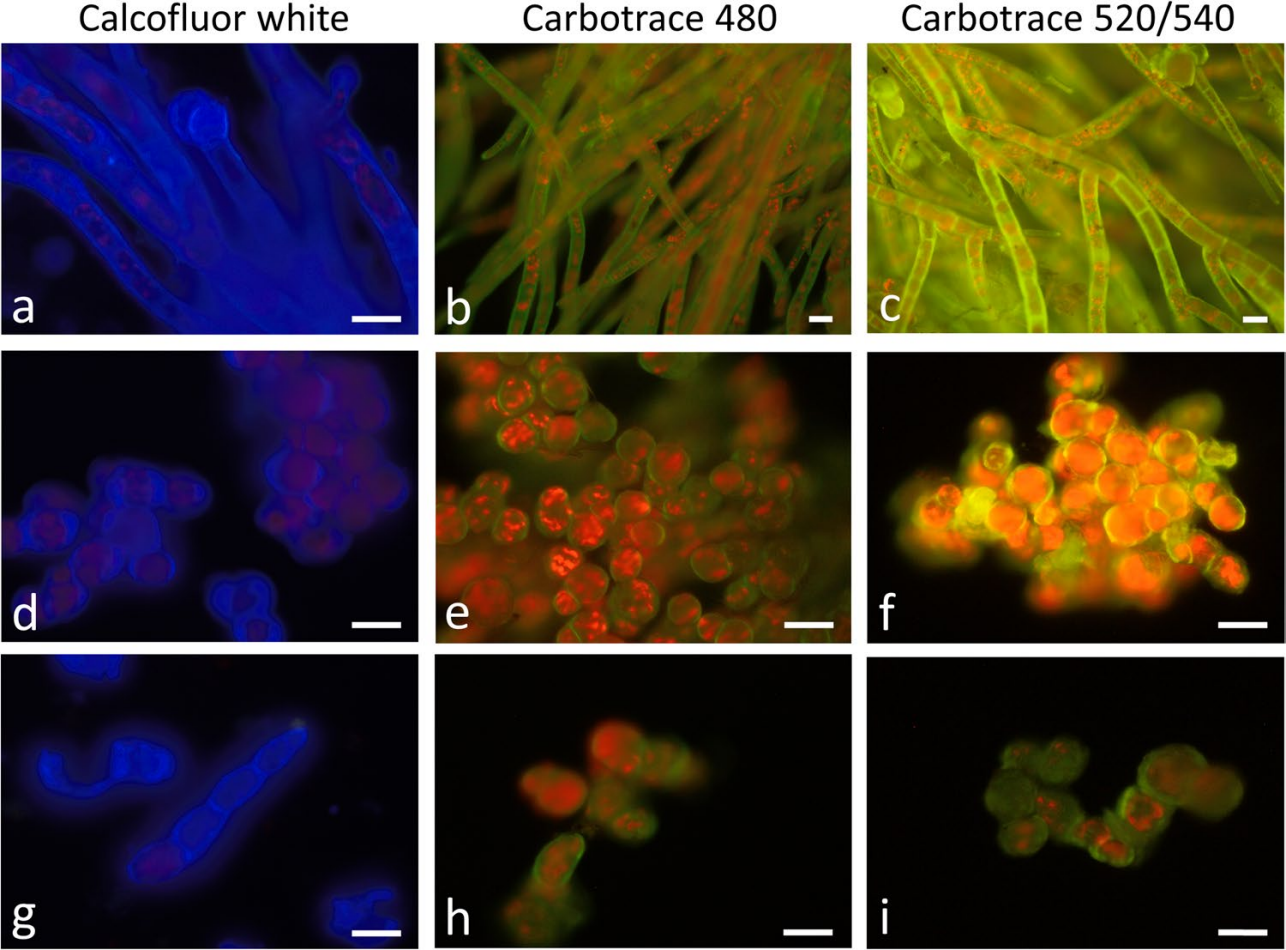
Cell wall stainings in *Trentepohlia aurea* (**a**–**c**), *Trentepohlia umbrina* (**d**–**f**), and *Trentepohlia jolithus* (**g**–**i**). Cells walls were stained with 1% calcofluor white (**a**, **d**, **g**) or Carbotrace 480 (**b**, **e**, **h**), Carbotrace 520 (**c**,** i**) and Carbotrace 540 (**f**). Scale bars 20 µm

The viability of the different *Trentepohlia* strains was tested by Auramine O, DIOC_6_, and FM 1–43 staining (Fig. 2). *Trentepohlia aurea* cells readily stained with Auramine O (Fig. 2a). After DIOC_6_ (Fig. 2b) and FM 1–43 (Fig. 2c) treatment, mostly the plasma membranes were stained. Staining with Nile red allowed to depict individual lipid droplets within a filament (Fig. 2d). In *T. umbrina* (Fig. 2e–g) and *T. jolithus* (Fig. 2i–k), the vital staining gave similar results, and DIOC_6_ and FM 1–43 resulted in a clear staining of the plasma membrane. Nile red stained several areas in *T. umbrina* (Fig. 2h) and in *T. jolithus* sometimes almost the entire cells were stained indicating a high abundance of lipid droplets (Fig. 2l). To visualize the cell walls of *T. aurea* (Fig. 3a–c), *T. umbrina* (Fig. 3d–f), and *T. jolithus* (Fig. 3g–i), we used calcofluor white (Fig. 3 a, d, g) which gave a strong signal in all three investigated species. The newly developed Carbotrace 480, 520, and 540 optotrackers for carbohydrates were tested for the first time in *Trentepohlia* and gave very good results in cell wall stainings in *T. aurea* (Fig. 3b, c), *T. umbrina* (Fig. 3e, f), and *T. jolithus* (Fig. 3h, i). Basicall all applied optotrackers gave similar results; however, the staining appeaed strongest with Carbotrace 540 (Fig. 3 f).

### Biochemical traits suggest differences between the investigated *Trentepohlia *species

**Fig. 4:**
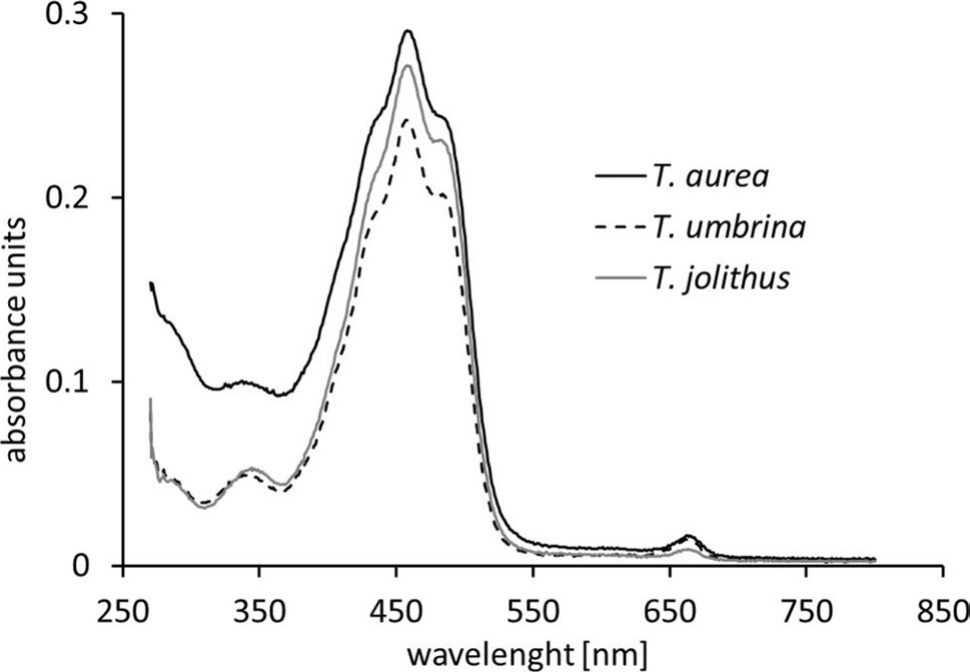
Absorbance spectra at different wave lengths of *Trentepohlia aurea*, *Trentepohlia umbrina*, and *Trentepohlia jolithus*

A remarkable optical feature of *Trentepohlia* is its intense orange to red coloration, which also stains the substrate macroscopically (Fig. 1a, d, g). A full spectrum of pigments revealed that mainly carotenoids are the reason for this coloration (Fig. 4). The ratio between chlorophyll *a* and carotenoids was highest in *T. jolithus* (15.9 carotenoid: Chl *a*) and similar between *T. aurea* (7.8) and *T. umbrina* (6.6).

The content of C and N differed between the three *Trentepohlia* species with *T. umbrina* exhibiting the highest content of 47% C and 2% N per dried biomass (Table 1). The molar C:N ratio was significantly higher in *T. aurea* with 28 compared to *T. umbrina* (20 C:N ratio) and *T. jolithus* (18 C:N ratio).

**Table 1:** N and C content in percent of field material from three *Trentepohlia* species. Letters indicate significant differences among the three species based on ANOVA followed by Tukey post hoc analysis

	N [%]	C [%]	C/N ratio
*T. aurea*	1.14^a^	32.30^a^	28.39^a^
*T. umbrina*	2.34^b^	46.54^b^	20.03^b^
*T. jolithus*	1.64^c^	29.08^a^	17.72^b^

The significant differences have to be indicated as superscript letters

**Fig. 5:**
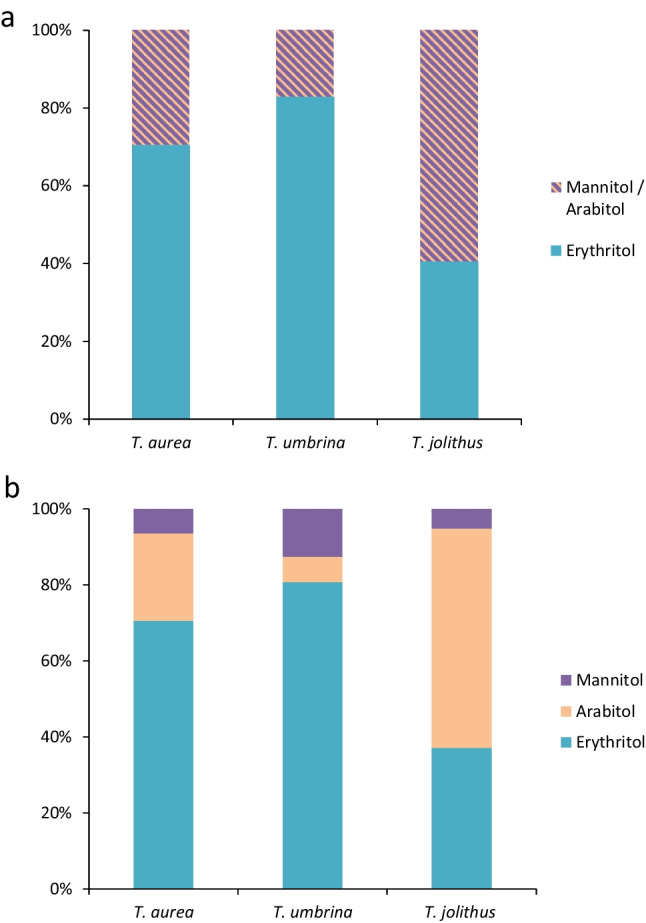
**a** Proportions between erythritol and mannitol/arabitol (indistinguishable) after high performance liquid chromatography (HPLC), **b** gas chromatography (GC)-derived proportions between mannitol, arabitol, and erythritol in *Trentepohlia aurea*, *Trentepohlia umbrina*, and *Trentepohlia jolithus*

By HPLC analysis, two compatible solutes were detected: erythritol and mannitol (Fig. 5a). *Trentepohlia umbrina* had the highest erythritol concentration, *T. jolithus* the highest mannitol concentration. In total, *T. aurea* showed the lowest concentration of compatible solutes and *T. jolithus* the highest concentration (Table 2).

**Table 2:** Calculated amount of compatible solutes (in µg per mg dry weight) derived from HPLC and GC analysis of field material from three *Trentepohlia* species

	Arabitol	Erythritol	Mannitol	Total polyols
*T. aurea*	6.77	19.66	1.74	28.17
*T. umbrina*	2.39	31.89	4.15	38.43
*T. jolithus*	25.87	18.31	2.10	46.28

Since the HPLC method did not separate all polyols, a GC analysis was undertaken, which allowed the separation of mannitol from arabitol. The GC analysis revealed that the HPLC “mannitol peak” in *T. aurea* and *T. jolithus* was dominated by arabitol (Fig. 5b). In contrast, in *T. umbrina* mannitol was quantitatively more important than arabitol (Fig. 5b).

### *Trentepohlia umbrina* and *Trentepohlia jolithus* showed recovery after desiccation

**Fig. 6:**
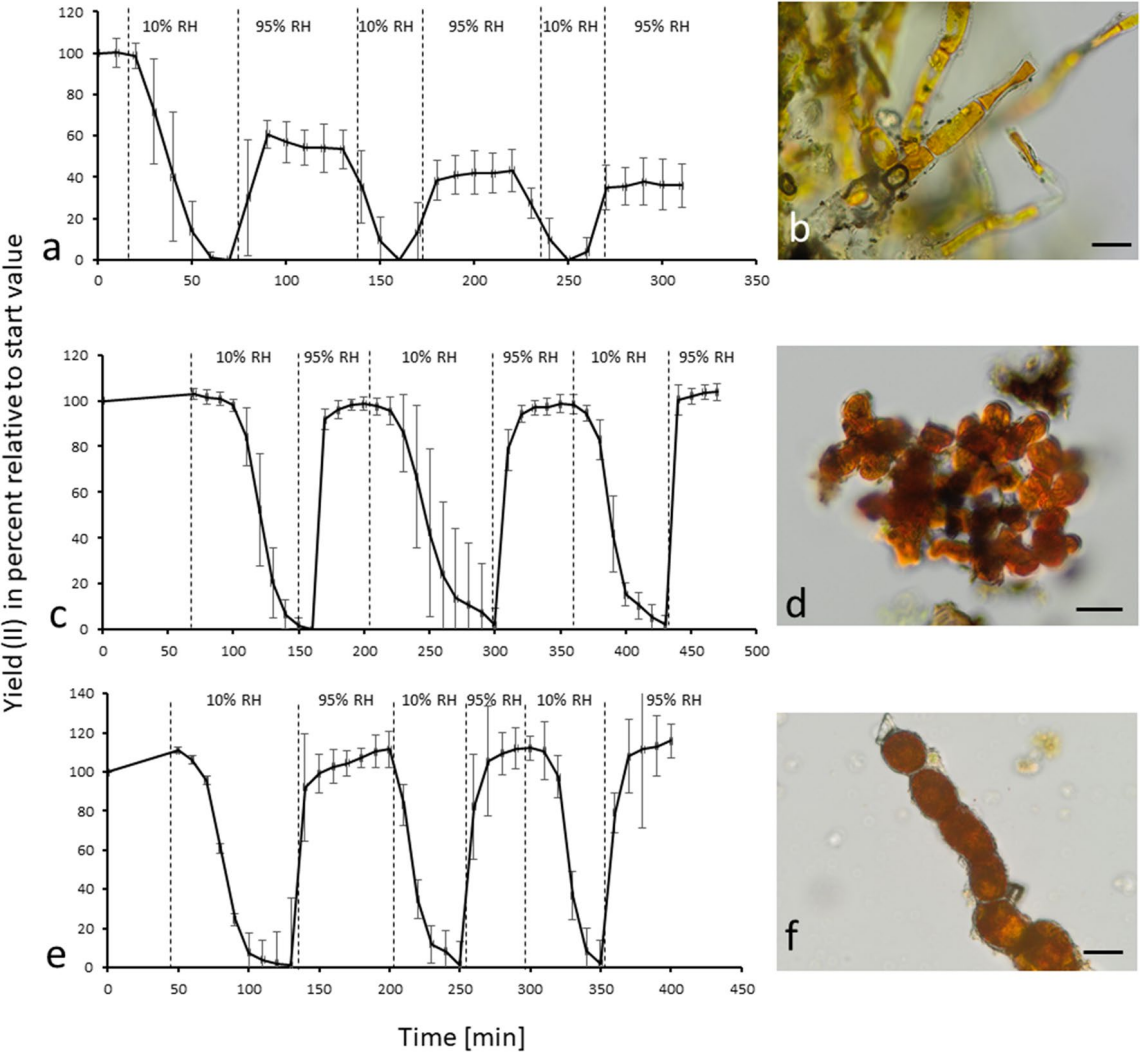
Desiccation rehydration curves (10% relative humidity (RH), rehydrated at 95% RH) and light microscopical images of desiccated samples of *Trentepohlia aurea* (**a**, **b**), *Trentepohlia umbrina* (**c**, **d**), and *Trentepohlia jolithus* (**e**, **f**). Each curve (**a**, **c**, **e**) shows 3 cycles of desiccation over silica gel followed by rehydration when the YII values had dropped to zero. Light micrographs were taken under immersion oil. Scale bars 20 µm

*Trentepohlia* biomass was transferred to a chamber with ~ 10% relative humidity and the photosynthetic potential (measured as effective quantum yield of PSII, YII) was constant for 1 to 2 h followed by a rapid decrease of YII to zero in all three investigated species (Fig. 6a, c, e). Figure 6 b (*T. aurea*), d (*T. umbrina*), and f (*T. jolithus*) illustrate the appearance of fully desiccated samples observed under immersion oil. At this point, the filters with the biomass were rewetted with BBM, but specimens were not directly soaked and *Trentepohlia* regained its photosynthetic potential: *T. umbrina* and *T. jolithus* immediately reached 100% of the photosynthetic capacity right before desiccation (Fig. 6c, e). This effect could be observed three times without a loss in recovery (Fig. 6c, e). *Trentepohlia aurea*, however, gained 50% of its initial photosynthetic potential also immediately after rehydrating (Fig. 6a). This could be repeated also three times in a row, always reaching nearly 50% of the initial value (Fig. 6a).

### Light curves indicated no photoinhibition in *Trentepohlia* sp. 

**Fig. 7:**
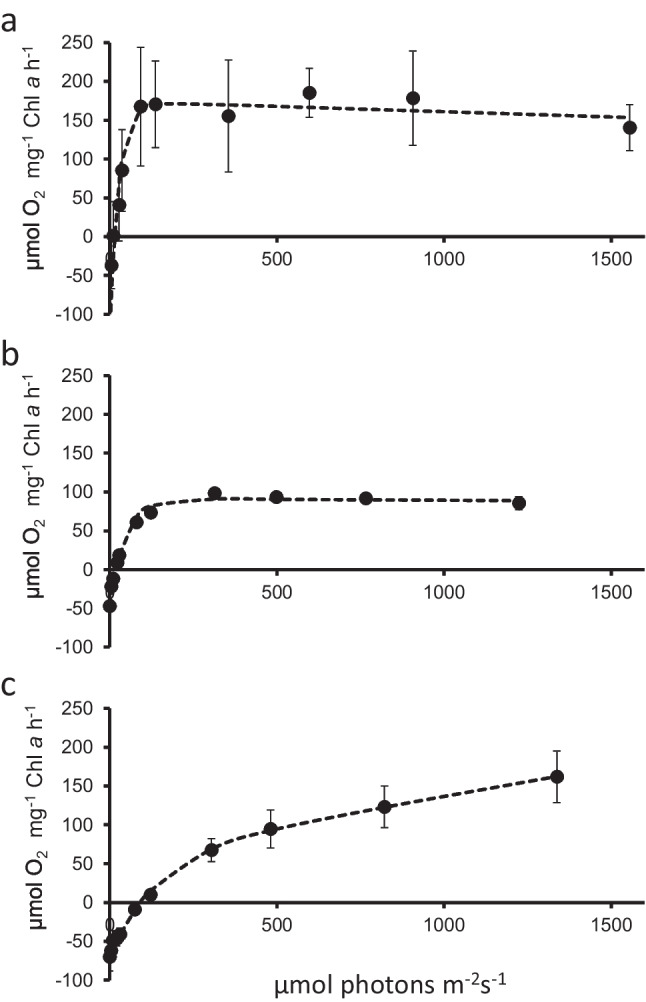
Photosynthesis irradiation (P-I) curves showing the oxygen production in µmol O_2_ mg^−1^ Chl *a* h^−1^ in dependence of increasing photon fluence rates in µmol photons m^−1^ s.^−1^ in *Trentepohlia aurea* (**a**), *Trentepohlia umbrina* (**b**), and *Trentepohlia jolithus* (**c**) (*n* = 4). Dotted line represents the fitted values according to Walsby (1997)

All three *Trentepohlia* species did not show photoinhibition (Fig. 7a–c); for *T. jolithus*, the curve did not even reach the saturation at ~ 1400 µmol photons m^−2^ s^−1^ (Fig. 7 c). *Trentepohlia umbrina* (Fig. 7b) and *T. jolithus* (Fig. 7c) were characterized by a low alpha value, which indicates a slow increase of oxygen evolution at low irradiance (Table 3).

**Table 3:** Parameters of photosynthesis-irradiance-curve after fitting using model by Walsby (1997); letters indicate significant differences between the species

	*T. aurea*	*T. umbrina*	*T. jolithus*
p_max_	300.1^a^	137.8^b^	130.4^b^
Alpha	11.39^a^	3.04^b^	0.93^b^
Respiration	− 129.03^a^	− 46.95^b^	− 70.12^b^
Ik	26.42^a^	45.27^a^	140.08^b^
Ic	14.78^a^	18.87^a^	92.57^b^

The significant differences have to be indicated as superscript letters

### Temperature curves suggest a broad temperature tolerance in all *Trentepohlia* sp. 

**Fig. 8:**
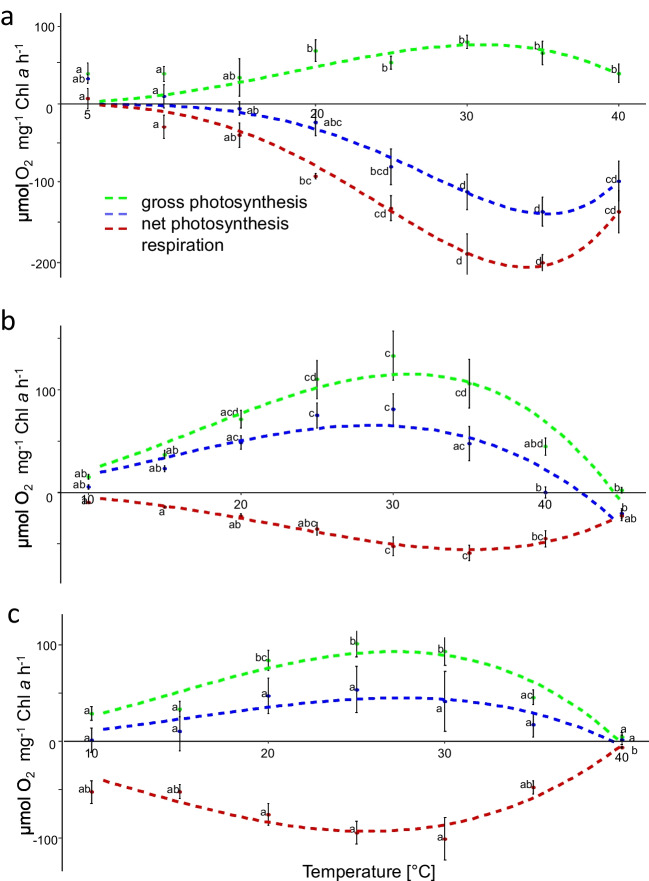
Temperature curves. Oxygen production measured in *Trentepohlia aurea* (**a**), *Trentepohlia umbrina* (**b**), and *Trentepohlia jolithus* (**c**) along a temperature gradient (*n* = 4). Dotted lines represent the fit after Yan and Hunt (1999). Green, gross-photosynthesis; blue, net-photosynthesis; red, respiration

All *Trentepohlia* species showed a broad temperature tolerance ranging from 5 to 40 °C with optimum photosynthesis between 20 and 35 °C (Fig. 8, Table 4). The gross-photosynthesis was similar with around 100 µmol O_2_ mg^−1^ Chl *a* h^−1^ for *T. umbrina* and *T. jolithus* and slightly lower in *T. aurea* (77 µmol O_2_ mg^−1^ Chl *a* h^−1^). The highest respiration rates were measured at slightly higher temperatures compared to photosynthesis at 30 to 35 °C for *T. aurea* and *T. umbrina* while in *T. jolithus*, the respiration optimum was at a lower temperature (Table 4). *T. aurea* showed a negative net-photosynthesis, the other two species a positive net-photosynthesis. In total, photosynthetic oxygen production ceased in *T. jolithus* close to 40 °C, while in *T.aurea* and *T. umbrina*, maximum photosynthesis temperatures were 43.1 and 44.5 °C, respectively.

**Table 4:** Optimum, maximum, and optimum temperature range (= 80% of maximum rate) for gross-photosynthesis and respiration for three *Trentepohlia* species

	Gross-photosynthesis			Respiration		
	*T. aurea*	*T. umbrina*	*T. jolithus*	*T. aurea*	*T. umbrina*	*T. jolithus*
Optimum	31 °C	31 °C	27.1 °C	33.9 °C	35.1 °C	25.7 °C
Maximum	43.1 °C	44.5 °C	39.9 °C	43.5 °C	47.4 °C	40.3 °C
Optimum range	23.5–36.9 °C	22.9–37.5 °C	19.7–33.3 °C	27.6–38.7 °C	27.3–41.1 °C	17.6–32.6 °C
Maximum rate (µmol O_2_ mg^−1^ Chl *a* h^−1^)	76.86	115.04	92.71	− 210.3	− 55.2	− 93.5

## Discussion

In the present study, we comparatively characterized three different *Trentepohlia* species microscopically by vital cell staining indicative of physiologically active cells with intact plasma membranes, cell walls, and lipid droplets. Analysis of the carotenoid contents showed the highest carotenoid to Chl *a* ratio in *T. aurea*, polyol analysis detected arabitol, erythritol and mannitol as major compounds, which were found in different abundancies, but *T. aurea* had the lowest total polyol content. Repeated cycles of desiccation resulted in a recovery of 50% of the initial YII in *T. aurea*, whereas *T. umbrina* and *T. jolithus* showed 100% recovery. Concerning photosynthetic oxygen production *T. aurea* showed the highest P_max_ values, in contrast *T. jolithus* had the highest light compensation point (Ik). For temperature requirements, *T. jolithus* had the lowest temperature optima, whereas *T. aurea* and *T. umbrina* were most active in a higher temperature range. These data allow for the first time a complete and comparative ecophysiological characterization of three different *Trentepohlia* species from contrasting terrestrial habitats.

### Vital staining showed mostly intact cells and cell walls

The investigated field samples had an orange to red appearance and were stained readily with Auramine O, indicative of physiologically active cells as reported in streptohyte green algae (Davey 1989, Trumhová et al. 2019). In order to test for membrane integrity, DIOC_6_ and FM 1–43 stains were used, which resulted in membrane staining in all three species comparable to observations in streptophyte and chlorophyte green algae (Holzinger et al. 2011; Terlova et al. 2021). FM 1–43 is an amphiphilic styryl dye, which was used to dissect vesicle trafficking in living plant cells (Bolte et al. 2004), and it can be also used as a vital stain as it binds to lipids and therefore will primarily be seen along the plasma in intact algal cells (Holzinger et al. 2014). In contrast, DIOC_6_ was originally used as a general membrane stain, which readily stained mitochondria and ER in various green algae (Hayashi and Ueda 1989; Holzinger et al. 2011). In the present study, endomembranes were detected with DIOC_6_ in *T. aurea*, but in *T. umbrina* and *T. jolithus*, mostly the plasma membranes were stained. Cell wall staining with calcofluor white (e.g., Herburger and Holzinger 2016) and Carbotrace showed that the cells were surrounded by intact cell walls. Carbotrace 480 (blue), Carbotrace 520 (green), and Carbotrace 530 (yellow) are newly developed carbohydrate specific optotracers, which were applicable to detect cell walls in the three *Trentepohlia* species. All these cell biological staining techniques were for the first time applied to this terrestrial genus.

### High carotenoid content and visualization of lipid droplets in *Trentepohlia*

*Trentepohlia* can be easily detected in the field by its conspicuous orange to red coloration which is due to high carotenoid contents (e.g., Kharkongor and Ramanujam 2015; Mukherjee et al. 2010; Chen et al. 2015, 2016). We found the highest carotenoid: Chl *a* ratio (15.9) in *T. jolithus* and lower ratios of 7.8 in *T. aurea* and 6.6 in *T. umbrina*. Previously, in *T. umbrina*, carotenoid ratios of 3 to 7 were reported by Ellwood et al. (2021), and a ratio 6.5 by Vergeiner (2011). Higher plants such as *Beta vulgaris*, *Zea mays*, or *Helianthus annuus* typically exhibit carotenoid:Chl *a* ratios between 0.17 and 0.28 in their leaves (Penuelas et al. 1995), while the biotechnologically important and astaxanthin producing green alga *Haematococcus pluvialis* has in the green and red phase carotenoid:Chl *a* ratios of 0.72 and 14.66, respectively (Solovchenko et al. 2011).

A study on *T. odorata* measured a strong correlation between light intensity and the amount of carotenoids (Tan et al 1993), which is expected. Carotenoids have a dual function, i.e., absorbing specific wavelengths as antenna pigments and conducting energy transfer to the reaction centers, but also serving as photoprotective substances of the photosynthetic apparatus (Takaichi 2011). With the spectrophotometric measurements of this study, it was not possible to derive which carotenoids are present and what function they have. As already numerous studies on *Trentepohlia* carotenoid composition are available, we did not put an advanced focus on this topic. An abundant occurrence of lipid droplets was observed in all three *Trentepohlia* species after Nile red stainings. Nile red is a lipid staining commonly used in diatoms (Prelle et al. 2019, 2021) and green algae (Rumin et al. 2015). Lipid droplets are common in green algae and have been found in streptophyte green algae in increased abundancies after different stress scenarios (Dadras et al. 2022) or nitrogen depletion (Pichrtová et al. 2016). They had a similar composition as described in land plants (De Vries and Ischebeck 2020). The fatty acid composition in *Trentepohlia arborum* has been investigated by Chen et al. (2016), who found triacylglycerol (TAG) as the largest fraction in total lipids, composed of oleic acid (18:1), palmitic acid (16:0) and minor amounts of linoleic acid (18:1) and hexadecadienoic acid (16:2).

### *Trentepohlia* shows high desiccation tolerance correlating with high polyol contents

In the present study we subjected *Trentepohlia* for the first time to 3 repeated cycles of strong desiccation over silica gel, leading to RHs of ~ 10% and monitored the photosynthetic yield (YII) as a proxy of physiological viability as previously applied to different algal species, at varying RHs (e.g., Karsten et al. 2014; Aigner et al. 2020; Terlova et al. 2021). While *T. aurea* recovered to only about 50% of the original YII values, *T. umbrina* and *T. jolithus* readily recovered to 100% of the initial value, even after 3 cycles of desiccation. *Trentepohlia aurea* was already described as a desiccation tolerant alga with a behavior “similar to gelatine” and it “dries out much slower than wool” (Howland 1929). Ong et al. (1992) performed desiccation experiments in *T. odorata* at 75% and 43% RH, which resulted in a decrease of photosynthesis activity at 43% RH. Treating *T. odorata* with 100% RH substantially increased P_max_ values, which was partially explained by a decrease in chlorophyll content. As already mentioned in the introduction Zhang et al. (2016, 2020) investigated the desiccation tolerance of a different isolate of *T. jolithus* from the west plateau klint of the Sichuan Basin, China. These authors followed various photophysiological parameters during desiccation and rewetting, and considered the reversible down-regulation of reaction center functions in PSII together with the maintenance of a basic linear electron flow during desiccation as essential traits for high desiccation tolerance. The rapid recovery capability of the original linear electron flow of desiccated algal cells contributes to this tolerance (Zhang et al 2016, 2020).

Ritchie and Heemboo (2021) concluded from the observation that photosynthesis of *Trentepohlia* was unaffected by pH (leading to a CO_2_ drop from 477 mmol m^−3^ at pH 5 to less than 1 mmol m^−3^ at pH 9) that they can facultatively use both CO_2_ and HCO_3_^−^ as inorganic carbon source. The same authors also tested the effect of increased osmolality and subjected *Trentepohlia* sp. to mannitol solutions of up to 1.508 Osmol kg^−1^, which resulted even in an increase of ETR_max_. This suggests that *Trentepohlia* has an incredible ability to tolerate water loss by desiccation or osmotic changes. Therefore, desiccation tolerance can be regarded as a central trait of *Trentepohlia* to survive in the terrestrial habitats where it was collected. *Trentepohlia aurea* covered the surface of limestones and *T. jolithus* grew abundantly on a concrete wall, habitats that were not shaded by vegetation and are likely exposed to evaporation, which quickly occurs when sunlight hits the surface. The growing site of *T. umbrina* can be regarded as a bit more protected, due to shading by the tree and the bark as organic substrate. *Trentepohlia umbrina* and *T. jolithus* recovered rapidly to 100% of the original YII after rehydration, which indicates that at least these species can use water nearly instantly after rewetting of the surface for photosynthesis. In contrast, *T. aurea* recovered only to 50% of the initial value.

But how is such a strong desiccation tolerance possible? There are early findings on the occurrence of low molecular weight carbohydrates like polyols in *Trentepohlia* (Feige and Kremer 1980). We could confirm the occurrence of arabitol, mannitol and erythritol by GC analysis, and these compounds occurred in different proportions. While erythritol was the most abundant polyol in *T. umbrina* and *T. aurea*, in *T. jolithus* arabitol dominated (Fig. 5). These carbohydrates act as compatible solutes and are important protective compounds against desiccation induced stress (e.g., Gustavs et al. 2010). The three polyols, however, exhibit different chemical properties and energy requirements for biosynthesis. Erythritol is the smallest polyol with 4 carbon atoms, while arabitol and mannitol consist of 5 and 6 C atoms, respectively. In addition, *T. umbrina* and *T. jolithus* showed similar molar C:N ratios (17.7–20), while that of *T. aurea* was with 28.4 much higher. *Trentepohlia aurea* has previously been shown to actively remove nitrogen (nitrate, nitrite, and ammonium) from water bodies (Abe et al. 2002), so when growing terrestrially, it might get nitrogen limited. According the Redfield stoichiometry (Redfield 1958), for phytoplankton, an enhanced C:N ratio is considered as indicative for nitrogen deficiency or even limitation. In addition, while the alpine sampling site can be regarded as rather pristine concerning atmospheric nitrogen deposition, the urban sites are usually characterized by nitrogen-rich precipitation. Taken together, we speculate that the alpine species was nutrient-limited compared to the other taxa, and hence unable to synthesize and accumulate higher concentrations of polyols. Consequently, the lower polyol content in *T. aurea* might explain the reduced desiccation tolerance.

### Photosynthesis in dependence of radiation and temperature shows a broad adaptation

Not much is known about the response of *Trentepohlia* towards increasing radiation. The three *Trentepohlia* species investigated in the present study showed a positive oxygen production up to ~ 1500 µmol photons m^−2^ s^−1 ^and none of the species showed photoinhibition. The P_max_ and alpha values were significantly higher in *T. aurea*, when compared to the other two species, indicating that this species had a very broad light tolerance, already capable of using low photon fluence rates for photosynthesis but also higher light did not limit the photosynthetic oxygen production. Similar observations were made in terrestrial *Klebsormidium* (Karsten et al. 2010). The other two investigated *Trentepohlia* sp. had lower P_max_ values and lower alpha, indicating that they were photophysiologically less active and particularly *T.* jolithus, with the lowest alpha value, was less effective at lower photon fluence rates as indicated by a significantly higher light compensation point (I_k_). Moreover, in the latter species, no plateau of the oxygen production was observed even at ~ 1400 µmol photons m^−2^ s^−1^, suggesting that even higher photon fluence rates could be used effectively for oxygen production. Taken together, these observations do not indicate that *Trentepohlia* species are shade-adapted. In contrast, another *Trentepohlia* sp. was described as shade-adapted by PAM measurements, with a very low optimum irradiance (E_opt_) of about 90 μmol photons m^−2^ s^−1^, and ETR_max_ about 80 μmol e^−^ g^−1^ chlorophyll-*a* s^−1^ (Ritchie and Heemboo 2021). For *T. odorata*, a low alpha value was reported, indicative of efficient use of low photon fluence rates (Ong et al. 1992). The I_k_ value of *T. odorata* (Ong et al. 1992) was similar to what we reported in *T. jolithus*, in contrast *T. aurea* and *T. umbrina* had significantly lower I_k_ values. This means, that the latter two species can use low photon fluence rates much better. The growth habitats of *T. aurea* and *T. umbrina* (the limestone rock and the tree bark) are occasionally shaded, meaning that the efficient use of low light is beneficial for these species. In contrast, *T. jolithus* abundantly grows on a concrete wall which is not shaded resulting in potentially high radiation that reached the surface.

Concerning temperature tolerance, the investigated *Trentepohlia* species were physiologically active between 5 and 40 °C with an optimum range of photosynthesis between 20 and 35 °C. These observations show higher temperature optima as the optimal temperature reported for best growth and reproduction under culture condition between 10 and 15 °C in *T. aurea* and *T. jolithus* at a light dark regime of 16:8 h (Rindi and Guiry 2002). Nevertheless, the data indicate that net photosynthesis was unexpectedly negative in *T. aurea* in contrast to both other species with positive photosynthesis. At this stage we can only speculate that bacteria in the field sample of *T. aurea* grew to such a high abundance that they strongly influenced the oxygen signals by their respiration.

In the field, the broad temperature tolerance is beneficial for the global distribution of the investigated species. When attempting to optimize carotenoid production, temperatures of 5 °C were found most effective in four *Trentepohlia* species (Kharkongor 2021).

## Conclusions

With the presented data we contribute to a better understanding of the cell biology, ecophysiology and biochemistry of a terrestrial green algal genus with a conspicuous red to orange appearance and growing on specific substrata exposed directly to air. We found clear differences between the investigated species in desiccation tolerance, photosynthetic performance and temperature demands, as well as in biochemical traits. The alpine *T. aurea* had the highest photosynthetic activity with the highest P_max_ values and the lowest alpha values, indicating that effective light use was already possible at very low irradiances, without signs of photoinhibition at high irradiances. However, *T. aurea* was the least desiccation tolerant of the investigated strains. The species differed in their qualitative and quantitative polyol composition, which represent important protective compounds against various stress conditions. But it remains ambiguous if the observed species-specific response patterns relate to the alpine or coastal collection sites.

## References

[CR1] Abe K, Imamaki A, Hirano M (2002). Removal of nitrate, nitrite, ammonium and phosphate ions from water by the aerial microalga *Trentepohlia aurea*. J Appl Phycol.

[CR2] Aigner S, Glaser K, Arc E, Holzinger A, Schletter M, Karsten U, Kranner I (2020). Adaptation to aquatic and terrestrial environments in *Chlorella vulgaris* (Chlorophyta). Front Microbiol.

[CR3] Bartoli F, Ellwood NTW, Bruno L, Ceschin S, Rugnini L, Caneva G (2019). Ecological and taxonomic characterisation of *Trentepohlia umbrina* (Kützing) Bornet growing on stone surfaces in Lazio (Italy). Ann Microbiol.

[CR4] Bolte S, Talbot C, Boutte Y, Catrice C, Read ND, Satiat-Jeunemaitre B (2004). FM-dyes as experimental probes for dissecting vesicle trafficking in living plant cells. J Microsc Oxford.

[CR5] Buschmann H, Zachgo S (2016). The evolution of cell division: from Streptophyte algae to land plants. Trends in Plants Science.

[CR6] Chapman RL, Borkhsenious O, Brown RC, Henk MC, Waters DA (2001). Phragmoplast-mediated cytokinesis in *Trentepohlia*: results of TEM and immunofluorescence cytochemistry. Int J System Evol Mirobiol.

[CR7] Chen L, Zhang L, Zhang W, Liu T (2015). Comparative analysis of growth and carotenoid accumulation of *Trentepohlia arborum* in aerial, subaerial, and aquatic cultivation. J. Appl Phycol.

[CR8] Chen L, Zhang L, Liu T (2016). Concurrent production of carotenoids and lipid by a filamentous microalga *Trentepohlia arborum*. Biores Technol.

[CR9] Dadras A, Fürst-Jansein JMR, Darienko T, Krone D, Scholz P, Riesenberg TP (2022). Environmental gradients reveal stress hubs predating plant terrestrialization. BioRxiv.

[CR10] Davey MC (1989). The effects of freezing and desiccation on photosynthesis and survival of terrestrial Antarctic algae and cyanobacteria. Polar Biol.

[CR11] De Vries J, Ischebeck J (2020). Ties between stress and lipid droplets pre-date seeds. Trends Plant Sci.

[CR12] Ellwood NTW, Bruno L, Caneva G (2021). Photosynthetic response to different light exposures and associated environmental conditions of the subaerial, epilithic green alga *Trentepohlia umbrina* (Chlorophyta, Ulvophyceae). Phycologia.

[CR13] Ettl H, Gärtner G (2014) Syllabus der Boden-, Luft- und Flechtenalgen, 2 edn. Springer Spectrum, p 777. 10.1007/978-3-642-39462-1

[CR14] Feige GB, Kremer BP (1980). Unusual carbohydrate pattern in *Trentepohlia* species. Phytochemistry.

[CR15] Garcia-Florentino C, Maguregui M, Morillas H, Marcaida I, Salcedo I, Madariaga JM (2018). *Trentepohlia* algae biofilms as bioindicator of atmospheric metal pollution. Sci Total Environm.

[CR16] Gustavs L, Eggert A, Michalik D, Karsten U (2010). Physiological and biochemical responses of green microalgae from different habitats to osmotic and matric stress. Protoplasma.

[CR17] Hametner C, Stocker-Wörgötter E, Rindi F, Grube M (2014) Phylogenetic position and morphology of lichenized Trentepohliales (Ulvophyceae, Chlorophyta) from selected species of Graphidaceae. 62: 170–186. 10.1111/pre.12055

[CR18] Hayashi Y, Ueda K (1989). The shape of mitochondria and the number of mitochondrial nucleoids during the cell cycle of *Euglena gracilis*. J Cell Sci.

[CR19] Herburger K, Holzinger A (2016). Aniline blue and calcofluor white staining of callose in the streptophyte green algae *Zygnema* and *Klebsormidium*. Bio-Protocols.

[CR20] Holzinger A, Lütz C, Karsten U (2011). Desiccation stress causes structural and ultrastructural alterations in the aeroterrestrial green alga *Klebsormidium crenulatum* (Klebsormidiophyceae, Streptophyta) isolated from an alpine soil crust. J Phycology.

[CR21] Holzinger A, Kaplan F, Blaas K, Zechmann B, Komsic-Buchmann K, Becker B (2014). Transcriptomics of desiccation tolerance in the streptophyte green alga *Klebsormidium* reveal a land plant-like defense. PLoS ONE.

[CR22] Howland LJ (1929). The moisture relations of terrestrial algae IV. Periodic observations of *Trentepohlia aurea* Martius. Ann Bot.

[CR23] Karsten U, Lütz C, Holzinger A (2010). Ecophysiological performance of the aeroterrestrial green alga *Klebsormidium crenulatum* (Charophyceae, Streptophyta) isolated from an alpine soil crust with an emphasis on desiccation stress. J Phycology.

[CR24] Karsten U, Herburger K, Holzinger A (2014). Dehydration, temperature and light tolerance in members of the aeroterrestrial green algal genus *Interfilum* (Streptophyta) from biogeographically different temperate soils. J Phycol.

[CR25] Kharkongor D (2021). Effect of photon irradiance and temperature on carotenoids accumulation in four species of *Trentepohlia* (Trentepohliales, Chlorophyta). Int J Complement Alt Med.

[CR26] Kharkongor D, Ramanujam P (2015) Spatial and temporal variation of carotenoids in four species of *Trentepohlia* (Trentepohliales, Chlorophyta). Journal of Botany. Article ID 201641, 10.1155/2015/20164

[CR27] Kjosen H, Arpin N, Liaaen-Jensen S (1972) Algal carotenoids. VI. The carotenoids of *Trentepohlia iolithus*. Isolation of β,β-carotene-2-ol, β,ε-carotene-2-ol and β,β-carotene-2,2‘-diol. *Acta Chemica Scandinavica* (1947–1973) 26(8): 3053–3067. 10.3891/acta.chem.scand.26-3053 4647667

[CR28] Klimesová M, Rindi F, Skaloud P (2019). DNA cloning demonstrates high genetic heterogeneity in populations of the subaerial green alga *Trentepohlia* (Trentepohliales, Chlorophyta). J Phycol.

[CR29] Köppen W, Geiger G (1930–1939). Handbuch der Klimatologie. 5 Bände, Gebrüder Borntraeger, Berlin 1930–1939

[CR30] Li Q, Liu J, Zhang L, Liu Q (2014). *De novo* transcriptome analysis of an aerial microalga *Trentepohlia jolithus:* pathway description and gene discovery for carbon fixation and carotenoid biosynthesis. PLOS one.

[CR31] Liu G, Zhang Q, Zhu H, Hu Z (2012). Massive Trentepohlia-bloom in a glacier valley of Mt. Gongga, China, and a new variety of Trentepohlia (Chlorophyta). PLoS ONE.

[CR32] López-Bautista JM, Waters DA, Chapman RL (2003). Phragmoplastin, green algae and the evolution of cytokinesis. Int J Syst Evol Microbiol.

[CR33] López-Bautista JM, Rindi F, Guiry MD (2006). Molecular systematics of the subaerial green algal order Trentepohliales: an assessment based on morphological and molecular data. Int J Syst Evol Microbiol.

[CR34] López-Bautista JM, Waters DA, Chapman RL (2002) The Trentepohliales Revisited. *Constancea* 83(1)

[CR35] Mukherjee R, Borah SP, Goswami BC (2010). Biochemical characterization of carotenoids in two species of *Trentepohlia* (Trentepohliales, Chlorophyta). J Appl Phycol.

[CR36] Nybraate G, Liaaen-Jensen S (1974). Algal carotenoids. 11. New carotenoid epoxides from *Trentepohlia iolithus*. Acta Chem Scand.

[CR37] Oliver MJ, Farrant JM, Hilhorst HWM, Mundree S, Williams B, Bewley D (2020). Desiccation tolerance: avoiding cellular damage during drying and rehydration. Ann Reve Plant Biol.

[CR38] Ong B-L, Lim M, Wee Y-C (1992). Effects of desiccation and illumination on photosynthesis and pigmentation of an edaphic population of *Trentepohlia odorata* (Chlorophyta). J Phycol.

[CR39] Penuelas J, Baret F, Filella L (1995). Semi-empirical indices to assess carotenoid/chlorophyll a ratio from leaf spectral reflectance. Photosynthetica.

[CR40] Pichrtová M, Arc E, Stöggl W, Kranner I, Hajek T, Hackl H, Holzinger A (2016). Formation of lipid bodies and changes in fatty acid composition upon pre-akinete formation in arctic and Antarctic *Zygnema* (Zygnematophyceae, Streptophyta) strains. FEMS Microbiol Ecol.

[CR41] Prelle LR, Graiff A, Gründling-Pfaff S, Sommer V, Kuriyama K, Karsten U (2019). Photosynthesis and respiration of Baltic Sea benthic diatoms to changing environmental conditions and growth responses of selected species as affected by an adjacent peatland (Hütelmoor). Front Microbiol.

[CR42] Prelle LR, Albrecht M, Karsten U, Damer P, Giese T, Jähns J, Müller S, Schulz L, Viertel L, Glaser K (2021). Ecophysiological and cell biological traits of benthic diatoms from coastal wetlands of the southern Baltic Sea. Front Microbiol.

[CR43] R Core Team. (2019) R: a language and environment for statistical computing. Vienna, Austria: R Foundation for Statistical Computing. http://www.R-project.org/. Accessed 15 June 2022

[CR44] Redfield AC (1958). The biological control of chemical factors in the environment. Am Sci.

[CR45] Rindi F, Guiry MD (2002). Diversity, life history, and ecology of *Trentepohlia* and *Printzia* (Trentepohliales, Chlorophyta) in urban habitats in western Ireland. J Phycol.

[CR46] Rindi F, López-Bautista JM (2007). New and interesting records of *Trentepohlia* (Trentepohliales, Chlorophyta) from French Guiana, including the description of two new species. Phycologia.

[CR47] Rindi F, Sherwood AR, Guiry MD (2005). Taxonomy and distribution of *Trentepohlia* and *Printzina* (Trentepohliales, Chlorophyta) in the Hawaiian Islands. Phycologia.

[CR48] Rindi F, Lam DW, López-Bautista JM (2009). Phylogenetic relationships and species circumscriptions in *Trentepohlia* and *Printzia* (Trentepohliales, Chlorophyta). Mol Phylogenet Evol.

[CR49] Ritchie RJ (2006). Consistent sets of spectrophotometric chlorophyll equations for acetone, methanol and ethanol solvents. Photosynth Res.

[CR50] Ritchie RJ, Heemboo M (2021). *Trentepohlia* sp., a terrestrial chlorophyte growing on galvanized iron lamp posts. Phycologia.

[CR51] Roach T, Böck N, Rittmeier N, Arc E, Kranner I, Holzinger A (2022). Acquisition of desiccation tolerance in *Haematococcus pluvialis* requires photosynthesis and coincides with lipid and astaxanthin accumulation. Algal Res.

[CR52] Rumin J, Bonnefond H, Saint-Jean B, Rouxel C, Sciandra A, Bernard O, Bougaran C-P, G,  (2015). The use of fluorescent Nile red and BODIPY for lipid measurement in microalgae. Biotechnol Biofuels.

[CR53] Solovchenko AE, Chivkunova OB, Maslova IP (2011). Pigment composition, optical properties, and resistance to photodamage of the microalga *Haematococcus pluvialis* cultivated under high light. Russian J Plant Physiol.

[CR54] Takaichi S (2011). Carotenoids in algae: Distributions, biosyntheses and functions. Mar Drugs.

[CR55] Tan CK, Lee YK, Ho KK (1993). Effect of light intensity and ammonium-N on carotenogenesis of *Trentepohlia odorata* and *Dunaliella bardawil*. J Appl Phycol.

[CR56] Terlova EF, Holzinger A, Lewis LA (2021). Terrestrial green algae show higher tolerance to dehydration than do their aquatic sister-species. Microb Ecol.

[CR57] Tischer J (1936). Über die Carotinoide und die Bildung von Jonon in Trentepohlia nebst Bemerkungen über den Gehalt dieser Alge an Erythrit (Carotinoide der Süßwasseralgen, II. Teil). Hoppe-Seyler’s Zeitschrift für physiologische Chemie.

[CR58] Trumhová K, Holzinger A, Obwegeser S, Neuner G, Pichrtová M (2019). The conjugating green alga *Zygnema* sp. (Zygnematophyceae) from the Arctic shows high frost tolerance in mature cells (pre-akinetes). Protoplasma.

[CR59] Vergeiner M (2011). Charakterisierung und Quantifizierung sekundärer Inhaltsstoffe der aerophytischen Alge *Trentepohlia iolithus* (Trentepohliophyceae) aus den Tiroler Alpen.

[CR60] Walsby AE (1997). Numerical integration of phytoplankton photosynthesis through time and depth in a water column. New Phytol.

[CR61] Wellburn AR (1994). The spectral determination of chlorophylls *a* and *b*, as well as total carotenoids, using various solvents with spectrophotometers of different resolution. J Plant Physiol.

[CR62] Yan W, Hunt LA (1999). An equation for modelling the temperature response of plants using only the cardinal temperatures. Ann Bot.

[CR63] Zhang L, Li Y, Liu J (2016). Complete inactivation of photosynthetic activity during desiccation and rapid recovery by rehydration in the aerial microalga Trentepohlia jolithus. Plant Biol.

[CR64] Zhang L, Li Y, Liu J (2020). Photosynthetic characteristics of aerial microalga *Trentepohlia jolithus* during drying and rewetting cycles. Algal Research.

[CR65] Zopf WF (1892) Beiträge zur Physiologie und Morphologie niederer Organismen 1. Kessinger Pub CO.

